# Analysis of risk factors for prolonged postoperative chest tube drainage after uniportal video-assisted thoracoscopic surgery pulmonary resection

**DOI:** 10.3389/fsurg.2026.1742102

**Published:** 2026-02-03

**Authors:** Wu Weinian, Li Yongjun

**Affiliations:** Xinjiang 474 Hospital, Urumqi, Xinjiang Uygur Autonomous Region, China

**Keywords:** deep nodule resection, FEV1%, prolonged chest tube drainage, pulmonary resection, risk factors, stapler cartridges, uniportal VATS

## Abstract

**Objective:**

This study aimed to identify independent risk factors for Prolonged Postoperative Chest Tube Drainage (PPCTD) in patients undergoing uniportal video-assisted thoracoscopic surgery (VATS) pulmonary resection.

**Methods:**

A retrospective observational cohort study was conducted involving 185 patients who underwent uniportal VATS pulmonary resection between January 2022 and December 2024. Patients were categorized into a prolonged drainage group (>7 days, *n* = 47) and a non-prolonged drainage group (≤7 days, *n* = 138) based on postoperative chest tube duration. Univariate and multivariate logistic regression analyses were performed to identify independent risk factors for PPCTD.

**Results:**

Multivariate analysis identified five independent risk factors for PPCTD: deep nodule resection (OR = 6.625, 95% CI: 2.512–17.469, *P* = 0.004), preoperative FEV1% < 70% (OR = 5.590, 95% CI: 1.987–15.728, *P* = 0.005), use of ≥4 stapler cartridges (OR = 4.775, 95% CI: 1.689–13.498, *P* = 0.012), intraoperative blood loss ≥250 mL (OR = 4.064, 95% CI: 1.421–11.623, *P* = 0.024), and preoperative anemia (OR = 3.434, 95% CI: 1.152–10.238, *P* = 0.033). A combined predictive model incorporating these factors demonstrated excellent discriminative ability (AUC = 0.892).

**Conclusion:**

Deep nodule resection, impaired pulmonary function (FEV1% < 70%), extensive stapler use (≥4 cartridges), significant intraoperative blood loss (≥250 mL), and preoperative anemia are significant independent risk factors for PPCTD following uniportal VATS pulmonary resection. These findings highlight potential targets for preoperative optimization and refined surgical technique. However, the derived predictive model requires external validation in independent cohorts before it can be considered for routine clinical use.

## 1 Introduction

Video-Assisted Thoracoscopic Surgery (VATS) serves as the bedrock of minimally invasive thoracic surgery (1). Its application in the diagnosis and treatment of pulmonary nodules has significantly reduced surgical trauma and accelerated patient postoperative recovery (2). Within this technical framework, uniportal VATS represents a further refined minimally invasive approach (3). By integrating the observation port with the operating port, it achieves less bodily tissue injury and superior postoperative pain control, leading to its widespread clinical adoption and application in anatomical pulmonary resections such as wedge resections and segmentectomies (4).

Despite continuous technological advancements, Prolonged Postoperative Chest Tube Drainage (PPCTD) remains a common clinical management challenge following VATS (5). Given its high incidence and direct impact on patient hospitalization duration and healthcare resource consumption, this metric is often regarded as a core endpoint for assessing the quality of perioperative outcomes (6). Abnormal prolongation of drainage time is not only a direct cause of patient discomfort and increased hospitalization costs but also an independent risk factor for complications such as pleural infection, persistent air leak, and atelectasis, severely constraining the effective implementation of the Enhanced Recovery After Surgery (ERAS) protocol in the field of thoracic surgery (7, 8).

Currently, most scholarly discussions on risk factors for prolonged postoperative drainage originate from retrospective studies on multiportal VATS or lobectomy (9). However, uniportal VATS pulmonary resection possesses unique characteristics in terms of surgical access, establishment of the operating triangle, and strategies for resolving instrument conflict (10, 11). For instance, the coincidence of the visual and operational axes may increase the technical difficulty of exposing deep-seated lesions and precisely placing stapler cartridges. The impact of these specific technical challenges on postoperative wound healing and pleural fluid production has not been fully elucidated or quantitatively evaluated. Furthermore, the predictive efficacy of patient-specific physiological reserves, such as preoperative impaired lung function and nutritional risk, as modifiable clinical variables, for drainage time in the context of this specific technique remains to be confirmed (12).

Therefore, to address the aforementioned research gaps, this study aims to systematically evaluate the clinical data of patients undergoing uniportal VATS pulmonary resection through a retrospective cohort analysis, with the goal of thoroughly identifying the independent risk factors leading to PPCTD. The conclusions of this study are expected to provide a solid evidence-based foundation for risk stratification, predictive medical intervention, and the development of personalized enhanced recovery protocols tailored to this specific surgical technique.

## 2 Materials and methods

### 2.1 General information

This retrospective cohort study was conducted with the approval of the Ethics Committee of Xinjiang 474 Hospital. A total of 185 patients who underwent uniportal video-assisted thoracoscopic pulmonary resection between January 2022 and December 2024 were retrospectively analyzed. Based on the duration of postoperative chest tube drainage, they were categorized into two groups: the prolonged drainage group (>7 days, *n* = 47) and the non-prolonged drainage group (≤7 days, *n* = 138).The threshold of >7 days for defining prolonged drainage was adopted based on prior studies in VATS pulmonary resection literature, where it has been commonly used to reflect clinically meaningful prolongation associated with increased morbidity and resource utilization, and aligns with institutional clinical pathways for chest tube management^6,96,9. Notably, recent studies focusing on chest tube duration after VATS have similarly utilized this threshold to define prolonged drainage and to evaluate risk factors or predictive models. Therefore, the >7-day cutoff represents a clinically relevant and widely accepted benchmark for PPCTD in contemporary VATS practice. It is critical to distinguish PPCTD from prolonged air leak (PAL). PAL, typically defined as an air leak persisting beyond a certain postoperative day, was not independently measured or used as the primary endpoint in this study. PPCTD is a broader clinical endpoint that encompasses all reasons for continued chest tube necessity, including but not limited to air leak, significant pleural effusion, and clinical judgment.

The inclusion criteria were as follows: (1) age between 18 and 80 years; (2) pulmonary nodules identified on preoperative CT requiring surgical intervention, meeting at least one of the following criteria: nodule diameter ≥8 mm with a solid component >50%, volume doubling time <400 days during follow-up, presence of malignant radiographic features (e.g., lobulation, spiculation, or pleural indentation), or patient's strong preference for surgery; (3) underwent uniportal video-assisted thoracoscopic pulmonary resection (sublobar or lobar); (4) availability of complete clinical and pathological data.

The exclusion criteria were: (1) conversion to thoracotomy or addition of surgical ports; (2) receipt of neoadjuvant chemotherapy or radiotherapy prior to surgery; (3) confirmation of extensive pleural adhesions during surgery that could confound the assessment of postoperative drainage; (4) history of previous ipsilateral thoracic surgery; (5) development of postoperative bronchopleural fistula requiring secondary surgical intervention; (6) missing key clinical variables (e.g., incomplete drainage records).

### 2.2 Observation indicators

Data on the following variables were systematically collected:

1. Baseline and preoperative characteristics: age, gender, body mass index (BMI), smoking history, history of diabetes, history of chronic obstructive pulmonary disease (COPD), percent predicted forced expiratory volume in one second (FEV1%), and preoperative serum albumin level. Smoking history and a history of COPD were specifically recorded as they represent key modifiers of preoperative pulmonary function, aiding in the interpretation of FEV1% values.

2. CT imaging features: All preoperative CT images were independently reviewed by two experienced thoracic surgeons blinded to the patient grouping and outcomes. Discrepancies were resolved by consensus. A deep-seated nodule was defined as one with the shortest distance from its nearest edge to the visceral pleura exceeding 2 cm. Pleural indentation was defined as the presence of a linear strand connecting the nodule to the pleura accompanied by pleural retraction.

3. Surgery-related variables: Type of resection (sublobar resection or lobectomy), total operative time (from skin incision to wound closure), intraoperative blood loss (calculated as the sum of suction canister volume and gauze weight difference), number of stapler cartridges used, incidence of deep nodule resection (confirmed by correlating preoperative CT localization with a postoperative pathological resection depth >2 cm), and performance of pleural repair (defined as the intraoperative suturing or stapled closure of identifiable visceral pleural defects judged to be at significant risk for air leak, excluding the routine staple line itself).

4. Preoperative anemia: was defined as a hemoglobin concentration <130 g/L for men and <120 g/L for women, according to the World Health Organization (WHO) criteria. Hemoglobin levels were obtained from the most recent routine blood test prior to surgery, typically within 30 days. No patients were excluded due to missing hemoglobin data, as this was a mandatory component of the preoperative assessment protocol.

### 2.3 Postoperative chest tube management protocol

The primary endpoint (chest tube duration) was directly influenced by a standardized, institution-wide chest tube management protocol, applied uniformly to all patients in this study. The key elements were as follows:

- ***Drain characteristics:*** A 28-French (Fr) straight silicone chest tube was routinely placed in the operating room.
- ***System and assessment:*** Drainage systems were analog (non-digital). Air leak was assessed clinically at least every 8 h by inspecting for bubbling in the water-seal chamber during quiet breathing and cough.
- ***Suction and water-seal policy:*** A negative pressure of −20 cm H₂O was applied immediately postoperatively. Suction was discontinued (converted to water-seal) on postoperative day 1 if no active air leak was evident, regardless of fluid output.
- ***Removal criteria:*** The chest tube was removed when all of the following criteria were met for at least 24 h: (1) no air leak on water-seal; (2) serous or serosanguinous drainage with a volume <300 mL over the preceding 24 h; and (3) clinical and radiographic evidence of lung expansion.
- ***Discharge policy:*** Patients were not discharged with a chest tube *in situ*. Tube removal was a prerequisite for discharge planning.

### 2.4 Surgical technique and operative details

***Fissure and adhesion assessment:*** The completeness of the pulmonary fissure was visually assessed intraoperatively and categorized as complete, incomplete, or fused. The presence and severity of pleural adhesions were graded as none, mild (filmy, easily separable), moderate (dense, requiring sharp dissection), or severe (obliterating the pleural space).

***Use of sealants or reinforcement materials:*** The routine use of pleural sealants (e.g., fibrin glue, synthetic hydrogel) or staple line reinforcement materials (e.g., bovine pericardial strips) was not part of the standard surgical protocol during the study period. Their selective use in individual cases, if any, was recorded but was exceedingly rare and not analyzed as a separate variable due to negligible frequency.

***Stapler type:*** All pulmonary parenchymal transactions and vascular divisions were performed using endoscopic staplers from a single manufacturer, with cartridge selection (e.g., tissue thickness, staple height) based on the surgeon's assessment of tissue thickness at the transection site.

***Use of sealants or reinforcement materials:*** The routine use of pleural sealants (e.g., fibrin glue, synthetic hydrogel) or staple line reinforcement materials (e.g., bovine pericardial strips) was not part of the standard surgical protocol during the study period. Their selective use in individual cases, if any, was recorded but was exceedingly rare and not analyzed as a separate variable due to negligible frequency.

### 2.5 Statistical analysis

Statistical analyses were performed using SPSS Statistics version 27.0. Normally distributed continuous data are presented as mean ± standard deviation and were compared using the independent samples *t*-test. Categorical data are presented as numbers (percentages) and were compared using the Chi-square test or Fisher's exact test, as appropriate. Variables with a *P*-value < 0.1 in the univariate analysis were entered into a multivariate logistic regression model (forward stepwise method) to identify independent risk factors for prolonged drainage, with results expressed as odds ratios (OR) and 95% confidence intervals (CI). Model calibration, which measures the agreement between predicted probabilities and observed outcomes, was assessed using the Hosmer-Lemeshow goodness-of-fit test and visually represented with a calibration plot. When candidate predictors were highly correlated (e.g., Phi coefficient > 0.6), the variable with clearer clinical relevance to the surgical procedure and outcome was prioritized for inclusion in the multivariate model to avoid multicollinearity. A two-sided *P*-value < 0.05 was considered statistically significant.

## 3 Results

### 3.1 Participant characteristics and group allocation

**Figure 1:**
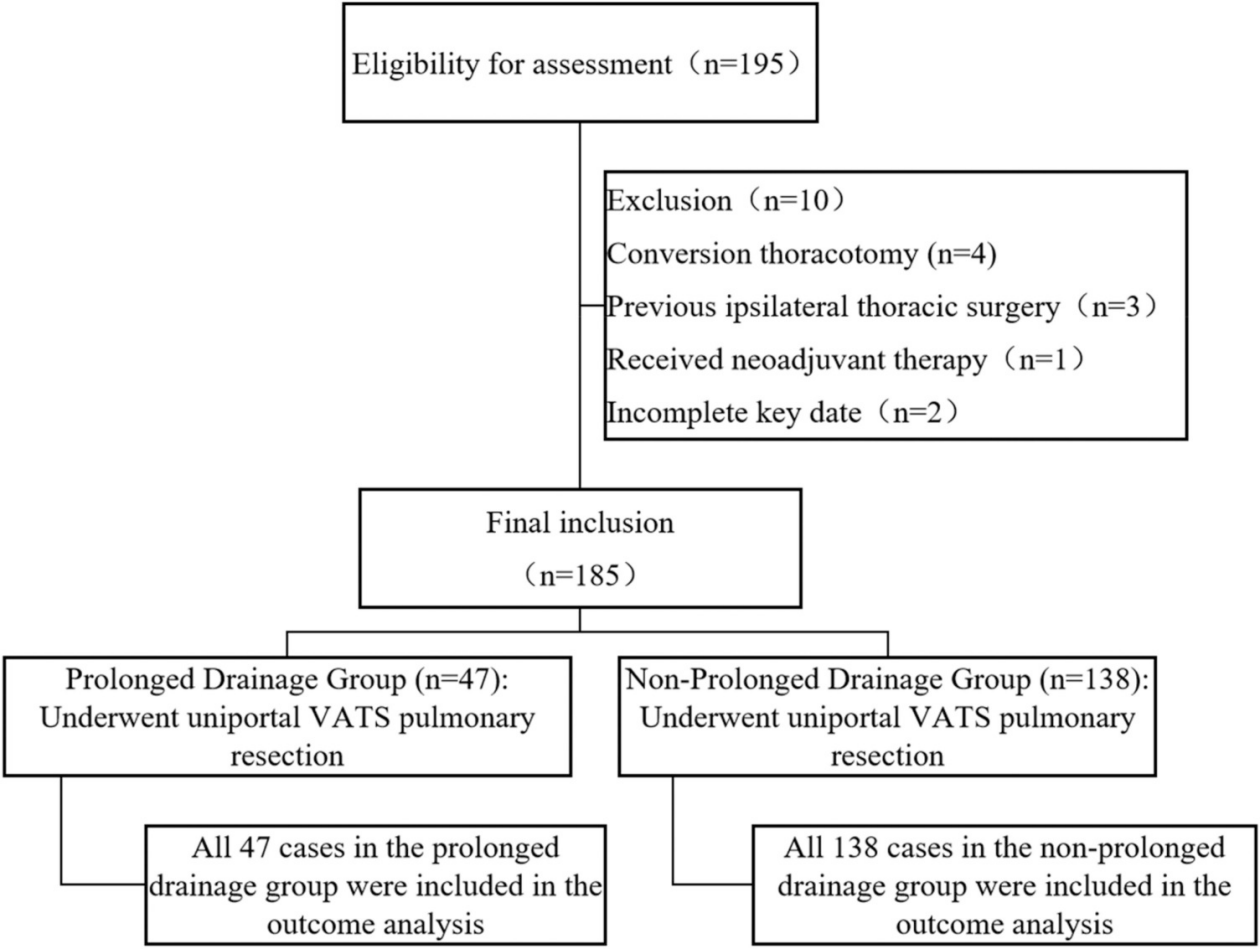
Flow chart of patient assignment.

Between January 2022 and December 2024, a total of 245 consecutive patients were initially screened for eligibility. Of these, 60 patients were excluded for the following reasons: conversion to thoracotomy or addition of surgical ports (*n* = 18); receipt of neoadjuvant therapy prior to surgery (*n* = 12); confirmation of extensive pleural adhesions during surgery (*n* = 10); history of previous ipsilateral thoracic surgery (*n* = 8); development of postoperative bronchopleural fistula requiring re-intervention (*n* = 5); and missing key clinical or drainage records (*n* = 7). Consequently, 185 patients who met the inclusion criteria and had complete data were included in the final analysis. Based on a postoperative drainage duration threshold of 7 days, they were categorized into a prolonged drainage group (*n* = 47) and a non-prolonged drainage group (*n* = 138). The patient selection process is also summarized visually in Figure 1. All enrolled patients completed the follow-up and were included in the final analysis without any loss to follow-up or missing data.

### 3.2 Univariate analysis of factors influencing prolonged drainage

Univariate analysis revealed statistically significant differences (*P* < 0.05) between the two groups regarding age, body mass index, history of diabetes, history of COPD, percent predicted FEV1%, preoperative albumin, preoperative anemia, deep-seated nodules, operative time, intraoperative blood loss, number of stapler cartridges used, deep nodule resection, and pleural repair (see Table 1). These variables were consequently included in the multivariate logistic regression analysis. In contrast, no significant differences (*P* > 0.05) were observed in gender, smoking history, pleural indentation sign, or surgical approach. The multivariate analysis ultimately identified deep nodule resection, use of ≥4 stapler cartridges, intraoperative blood loss ≥250 mL, FEV1% < 70%, and preoperative anemia as independent risk factors for prolonged postoperative chest tube duration.

**Table 1:** Comparison of baseline characteristics, CT features, and surgical indicators between the two groups.

Indicator	Prolonged Group (*n* = 47)	Non-Prolonged Group (*n* = 138)	Statistic	*P*-value	OR (95% CI)
Baseline Data					
Age (years, x¯ ± s)	68.1 ± 7.9	60.8 ± 9.2	*t* = 5.012	<0.001	-
Male [*n*(%)]	32 (68.1)	82 (59.4)	*χ*² = 1.205	0.272	1.46 (0.73–2.92)
BMI (kg/m², x¯ ± s)	26.5 ± 2.8	24.1 ± 2.9	*t* = 5.103	<0.001	-
Smoking history [*n*(%)]	25 (53.2)	60 (43.5)	χ²=1.456	0.228	1.48 (0.76–2.87)
History of diabetes [*n*(%)]	13 (27.7)	20 (14.5)	χ²=4.383	0.036	2.25 (1.03–4.94)
History of COPD [*n*(%)]	10 (21.3)	12 (8.7)	χ²=5.647	0.017	2.85 (1.15–7.05)
FEV1% predicted (x¯ ± s)	66.8 ± 8.5	80.2 ± 9.1	*t* = −8.941	<0.001	-
Preoperative albumin (g/L, x¯ ± s)	35.8 ± 3.5	40.5 ± 3.8	t = −7.617	<0.001	-
Preoperative anemia [*n*(%)]	11 (23.4)	15 (10.9)	χ² = 4.876	0.027	2.53 (1.07–5.99)
CT Features					
Deep-seated nodule [*n*(%)]	26 (55.3)	31 (22.5)	χ² = 18.714	<0.001	4.38 (2.23–8.61)
Pleural indentation sign [*n*(%)]	20 (42.6)	45 (32.6)	χ² = 1.656	0.198	1.53 (0.78–3.01)
Surgery-related Indicators					
Surgical approach [*n*(%)]			χ² = 2.783	0.095	
Sublobar resection	34 (72.3)	115 (83.3)			1.00 (Ref)
Lobectomy	13 (27.7)	23 (16.7)			1.91 (0.87–4.22)
Operative time (min, x¯ ± s)	148.6 ± 35.2	121.3 ± 29.7	*t* = 5.012	<0.001	-
Intraop. blood loss (mL, x¯ ± s)	278.5 ± 70.1	188.2 ± 58.4	*t* = 8.756	<0.001	-
Number of stapler cartridges (x¯ ± s)	4.6 ± 1.1	3.4 ± 0.9	*t* = 7.543	<0.001	-
Deep nodule resection [*n*(%)]	22 (46.8)	20 (14.5)	χ² = 22.115	<0.001	5.23 (2.51–10.91)
Pleural repair [*n*(%)]	15 (31.9)	14 (10.1)	χ² = 13.521	<0.001	4.20 (1.85–9.56)

Measurement data conforming to a normal distribution are presented as mean ± standard deviation and were compared using the independent samples *t*-test. Categorical data are presented as counts (percentages) and were compared using the χ² test. *P* < 0.05 was considered statistically significant. indicates a statistically significant difference (*P* < 0.05). BMI, body mass index.

Bold values indicate a statistically significant difference between groups (*P* < 0.05).

### 3.3 Multivariate logistic regression analysis of risk factors for prolonged chest tube duration

Multivariate logistic regression analysis revealed that deep nodule resection (OR = 6.625, *P* = 0.004), FEV1% < 70% (OR = 5.590, *P* = 0.005), use of ≥4 stapler cartridges (OR = 4.775, *P* = 0.012), intraoperative blood loss ≥250 mL (OR = 4.064, *P* = 0.024), and preoperative anemia (OR = 3.434, *P* = 0.033) were identified as independent risk factors for prolonged postoperative chest tube duration. In contrast, the associations of lobectomy (*P* = 0.090) and preoperative hypoalbuminemia (*P* = 0.073) with prolonged drainage did not reach statistical significance. To further assess the potential influence of resection type, a sensitivity analysis was performed by forcing the variable “lobectomy vs. sublobar resection” into the final multivariate model. The results remained consistent, and none of the identified independent risk factors changed materially, supporting the robustness of the primary findings across different resection types. The final multivariate logistic regression model is best understood as a perioperative risk prediction tool. It incorporates both preoperative (e.g., FEV1% < 70%, preoperative anemia) and intraoperative (e.g., deep nodule resection, ≥4 stapler cartridges, blood loss ≥250 mL) variables, allowing for updated risk stratification immediately after surgery to guide postoperative management.

### 3.4 Specification and prediction equation

The final multivariate logistic regression model for predicting prolonged chest tube drainage (>7 days) was specified as follows: Variables and Coding: All predictor variables were coded as binary (0 = “No” or absent, 1 = “Yes” or present). Specifically: “Deep nodule resection”, “Stapler cartridges used ≥4”, “Intraoperative blood loss ≥250 mL”, “FEV1% < 70%”, “Preoperative anemia”, “Lobectomy”, and “Preoperative albumin <35 g/L”. Coefficients: The intercept and *β* coefficients for each predictor are provided in Table 2. Linear Predictor (LP): The log-odds of PPCTD can be calculated as: LP = −4.112 + (1.891 × DeepNodule) + (1.563 × Stapler ≥ 4) +(1.402 × BloodLoss ≥ 250) + (1.721 × FEV1 < 70) + (1.234 × Anemia) + (0.985 × Lobectomy) + (1.101 × Albumin < 35). Predicted Probability (P): The probability of prolonged drainage is then obtained using the logistic function: *P* = e^LP^/(1 + e^LP^), where *e* is the base of the natural logarithm. This model can be used to compute individual patient risk based on pre- and intraoperative characteristics.

**Table 2:** Multivariate logistic regression analysis of risk factors for prolonged chest tube duration.

Risk Factor	*β*	Wald χ²	OR	95% CI	*P*
Intercept (Constant)	−4.112	15.237	-	-	<0.001
Deep nodule resection (Yes vs. No)	1.891	8.115	6.625	(2.512–17.469)	0.004
Stapler cartridges used ≥4 (Yes vs. No)	1.563	6.328	4.775	(1.689–13.498)	0.012
Intraoperative blood loss ≥250 mL (Yes vs. No)	1.402	5.112	4.064	(1.421–11.623)	0.024
FEV1% < 70% (Yes vs. No)	1.721	7.894	5.590	(1.987–15.728)	0.005
Preoperative anemia (Yes vs. No)	1.234	4.556	3.434	(1.152–10.238)	0.033
Lobectomy (Yes vs. No)	0.985	2.874	2.678	(0.891–8.045)	0.090
Preoperative albumin <35 g/L (Yes vs. No)	1.101	3.221	3.007	(0.912–9.912)	0.073

OR, odds ratio; CI, confidence interval.

### 3.5 Sensitivity analysis of predictive factors

Diagnostic efficacy analysis of the five independent risk factors revealed that the combined predictive model demonstrated the best performance [Area Under the Curve (AUC) = 0.892]. Preoperative FEV1% (AUC = 0.812) and intraoperative blood loss (AUC = 0.801) also exhibited excellent predictive ability. The number of stapler cartridges used showed good predictive value (AUC = 0.758), while deep nodule resection (AUC = 0.662) and preoperative anemia (AUC = 0.563) demonstrated relatively limited predictive performance. At their optimal thresholds, preoperative FEV1% < 70% showed balanced sensitivity (0.766) and specificity (0.783), while intraoperative blood loss≥250 mL demonstrated higher specificity (0.826). The use of ≥4 stapler cartridges showed higher sensitivity (0.745), making it suitable for initial screening. Although preoperative anemia had low sensitivity (0.234), its high specificity (0.891) suggests value in confirming high-risk cases when present.

Model stability was confirmed through Bootstrap resampling (1000 iterations), which showed that the 95% confidence intervals for all AUCs excluded 0.5. The cross-validation accuracy was 85.2%, closely matching the original model's accuracy of 86.1%, indicating good model generalizability and stability. The Hosmer-Lemeshow goodness-of-fit test for the combined model yielded a *χ*² value of 6.24 with a *P*-value of 0.342, suggesting no significant deviation from perfect calibration (*P* > 0.05). The calibration plot further demonstrates good agreement between predicted and observed probabilities across risk deciles. This analysis confirms that these predictors, particularly when combined, provide robust and well-calibrated identification of patients at risk for prolonged chest tube duration. This analysis confirms that these predictors, particularly when combined, provide robust identification of patients at risk for prolonged chest tube duration with substantial clinical application value. See Table 3.

**Table 3:** Diagnostic efficacy of predictors for prolonged chest tube duration.

Variables	AUC	Sensitivity	Specificity	Youden index	Optimal threshold
Deep Nodule Resection	0.662 (0.580–0.744)	0.468	0.855	0.323	-
Stapler Cartridges Used	0.758 (0.686–0.830)	0.745	0.710	0.455	≥4 cartridges
Intraoperative Blood Loss	0.801 (0.736–0.866)	0.702	0.826	0.528	≥250 mL
Preoperative FEV1%	0.812 (0.750–0.874)	0.766	0.783	0.549	<70%
Preoperative Anemia	0.563 (0.474–0.652)	0.234	0.891	0.125	-
Combined Model	0.892 (0.842–0.942)	0.830	0.812	0.642	-

AUC, area under the receiver operating characteristic curve. The Youden index (Sensitivity + Specificity-1) was used to determine the optimal predictive threshold for continuous variables. The combined model represents the integrative predictive value of all five independent risk factors identified in the multivariate analysis. Model performance was validated using Bootstrap resampling with 1,000 iterations.

Bold values indicate a statistically significant difference between groups (*P* < 0.05).

### 3.6 Assessment of correlation between related predictors

Prior to multivariate analysis, we assessed the potential overlap between the CT-defined “deep-seated nodule” and the surgically confirmed “deep nodule resection”. A strong correlation was found, with a Phi coefficient of 0.682 (*χ*² = 86.33, *P* < 0.001). This indicates substantial overlap, as expected, since a deeply located nodule on CT often necessitates a deep parenchymal resection. Given their conceptual and statistical linkage, and because “deep nodule resection” directly reflects the surgical act and its consequences, it was selected for inclusion in the multivariate model as the more surgically relevant and definitive variable.

### 3.7 Internal validation and calibration of the predictive model

**Figure 2:**
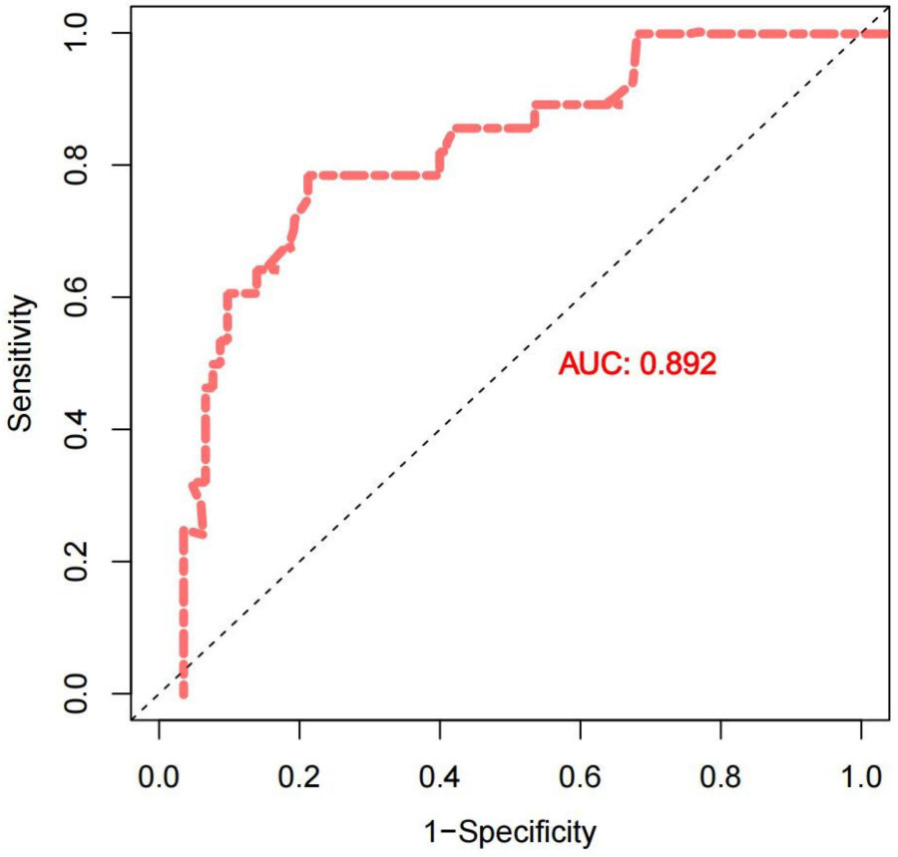
Receiver operating characteristic (ROC) curve of the combined predictive model.

**Figure 3:**
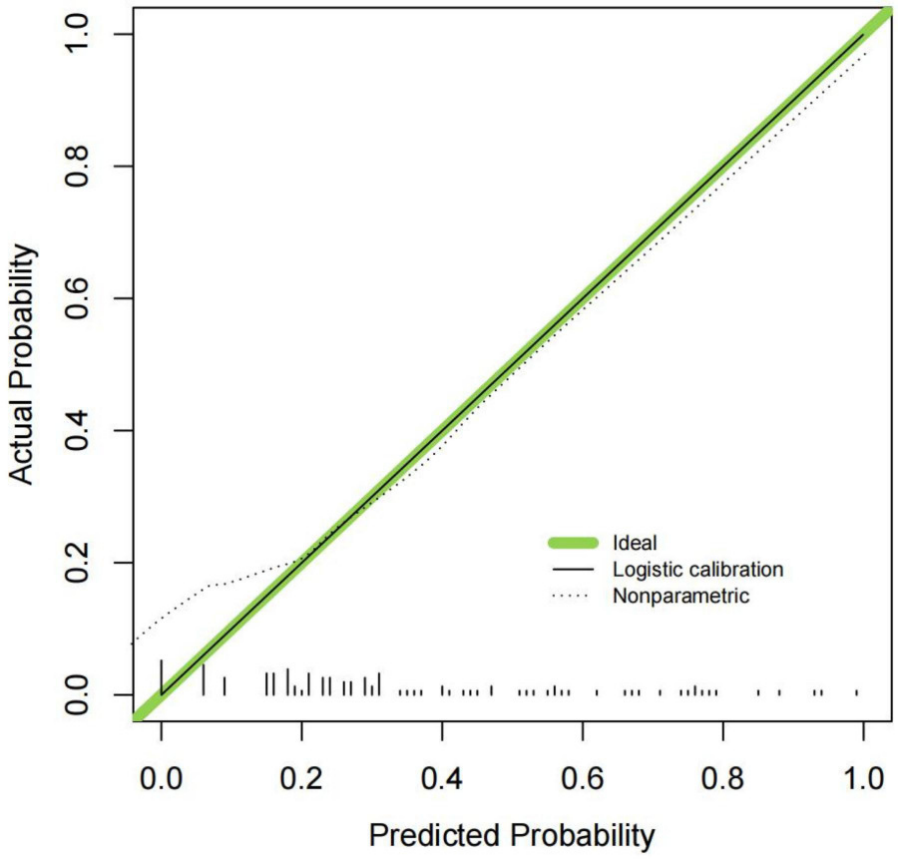
Calibration curve of prediction accuracy evaluation.

The combined predictive model demonstrated excellent discriminative ability, with an area under the receiver operating characteristic (ROC) curve (AUC) of 0.892 (95% CI: 0.842–0.942) (Figure 2). The model's calibration, which assesses the agreement between predicted probabilities and observed outcomes, was satisfactory. This was supported by a Hosmer–Lemeshow goodness-of-fit test (*χ*² = 6.24, degrees of freedom = 8, *P* = 0.342) and a low overall Brier score of 0.118. Visual inspection of the calibration plot (Figure 3) further confirmed good alignment along the line of perfect fit, with a calibration-in-the-large intercept of −0.03 (95% CI: −0.10 to 0.04) and a calibration slope of 0.95 (95% CI: 0.87 to 1.03).To evaluate model stability and potential overfitting, internal validation was performed via bootstrap resampling (1,000 iterations). This process involved refitting the entire model (including variable selection and coefficient estimation) in each bootstrap sample to estimate optimism. The optimism-corrected AUC was 0.879 (95% CI: 0.825–0.933), and the corresponding optimism-corrected calibration slope was 0.92. These results indicate that the model maintains robust performance with minimal overfitting in our cohort.

## 4 Discussion

This study focused on identifying independent risk factors for PPCTD following uniportal VATS pulmonary resection. Through retrospective analysis of clinical data from 185 patients, we identified deep nodule resection, FEV1% < 70%, use of ≥4 stapler cartridges, intraoperative blood loss ≥250 mL, and preoperative anemia as independent predictors of this complication. These findings not only elucidate the multifactorial mechanism underlying prolonged drainage in this specific surgical approach but also provide crucial evidence for clinical risk stratification and individualized patient management. It is important to explicitly state that chest tube duration, as the primary endpoint, is inherently influenced by institutional protocols (detailed in Section 2.4), suction strategy, drain system characteristics, and ultimately, clinician decision-making regarding removal. The lack of universal standardization in these practices means that the identified associations could be influenced by these center-specific factors, which is an inherent limitation of observational studies in this domain.

The multivariate logistic regression analysis clearly identified five independent risk factors: deep nodule resection (OR = 6.625), preoperative FEV1% < 70% (OR = 5.590), use of ≥4 stapler cartridges (OR = 4.775), intraoperative blood loss ≥250 mL (OR = 4.064), and preoperative anemia (OR = 3.434). These findings not only confirm the combined decisive role of surgical technical complexity and patients' baseline physiological status in postoperative recovery, but also highlight the unique weighting of certain risk factors specific to the uniportal approach. Although the type of resection (lobectomy vs. sublobar resection) did not emerge as an independent risk factor in our multivariate model, we acknowledge that different resection extents inherently carry varying risks of air leak and pleural fluid production. The comparable risk profile observed in our cohort may reflect the standardized technical approach and drain management protocol applied in our uniportal VATS program. Nevertheless, clinicians should remain aware that extensive resections such as lobectomy may still pose higher intrinsic risks, and individualized assessment is warranted.

Deep nodule resection was confirmed as the most significant independent predictor. This strong association relates directly to the inherent technical challenges of managing deep-seated lesions during uniportal VATS (13). Compared to the multi-angular visualization provided by multiportal approaches, the single incision in uniportal VATS leads to frequent instrument conflict and complete overlap of the visual and operative axes (14). These limitations substantially compromise the exposure, precise dissection, and optimal placement of stapler cartridges for deep nodules. To achieve complete resection of nodules located >2 cm from the visceral pleura, surgeons typically need to perform more extensive parenchymal dissection (15). This not only increases the risk of unplanned pulmonary tissue tearing or contusion but also creates a larger potential surface for leakage (16). Furthermore, during deep stapling procedures, increased tissue thickness and tension may compromise ideal cartridge formation, consequently elevating the probability of complications such as persistent alveolar air leak or increased tissue fluid exudation, which can contribute to prolonged drainage (17).

The use of ≥4 stapler firings was identified as another crucial independent risk factor. The number of stapler cartridges serves as an objective quantitative indicator of surgical extent and anatomical complexity (18). Increased cartridge usage directly corresponds to longer pulmonary parenchymal cutting and staple lines, which undoubtedly expands the potential surface area for postoperative air leakage (19). From a biomechanical perspective, longer staple lines are subjected to more complex tension distribution during lung re-expansion, where localized stress concentration may elevate the risk of staple line disruption (20). From a tissue response standpoint, repeated stapling may induce more pronounced local tissue compression, ischemia, and inflammatory response along the elongated staple line, which could collectively impair the regional microcirculation and consequently delay wound healing and re-epithelialization (21, 22). These findings strongly suggest that, while maintaining oncological radicality and surgical safety, optimizing resection planning and enhancing stapling efficiency to minimize stapler cartridge usage may serve as an important technical strategy for reducing drainage duration (23).

Intraoperative blood loss ≥250 mL, serving as a direct reflection of surgical trauma magnitude, demonstrates a strong association with PPCTD consistent with the logical pathophysiology. Substantial bleeding typically accompanies more extensive tissue dissection, denser adhesionlysis, or unanticipated vascular injury (24, 25). This degree of trauma triggers more intense local and systemic inflammatory responses, releasing abundant inflammatory mediators. These mediators not only increase systemic capillary permeability, promoting transudation of vascular fluid components into interstitial spaces and the pleural cavity, but may also directly inhibit the reparative function of pleural mesothelial cells. These combined effects ultimately lead to increased postoperative pleural drainage volume and prolonged drainage duration (26, 27).

Regarding patient-related factors, the significant predictive value of preoperative FEV1% < 70% underscores the crucial role of baseline pulmonary function in postoperative recovery (28). Impaired lung function frequently coexists with underlying pathological conditions such as chronic obstructive pulmonary disease (COPD) (29). The mechanisms leading to PPCTD in these patients are multifactorial: First, diminished lung elastic recoil and respiratory muscle strength contribute to inadequate postoperative lung re-expansion and increased residual pleural dead space, thereby impairing physiological absorption of pleural fluid (30). Second, effective cough capability is essential for clearing respiratory secretions and promoting lung reexpansion; patients with poor pulmonary function typically exhibit compromised cough efficiency, facilitating pleural fluid retention (31, 32). Furthermore, chronic hypoxemia may inhibit fibroblast proliferation and collagen synthesis and deposition, consequently delaying mechanical healing of pleural wounds (33). Finally, such patients often manifest a persistent chronic inflammatory state with elevated serum levels of pro-inflammatory cytokines (e.g., IL-6, TNF-α), and surgical trauma exacerbates this response, leading to more pronounced inflammatory exudation (34).

It is important to note that intraoperative variables such as blood loss ≥250 mL and the use of ≥4 stapler cartridges, while identified as independent risk factors in our model, may also serve as robust indicators of underlying surgical complexity. Significant bleeding often accompanies challenging dissections (e.g., in cases with dense adhesions or deep-seated lesions), and a high number of stapler firings directly reflects the extent of parenchymal transection and the technical demands of the resection. Thus, these factors likely represent both measures of procedural complexity and contributors to prolonged drainage through distinct pathophysiological pathways (e.g., inflammatory exudation or increased staple line surface area). This distinction cautions against interpreting them solely as direct causal agents but underscores their high utility as integrated, objective markers for intraoperative risk stratification. From a clinical perspective, their value lies in alerting the surgical team to cases with higher technical demands, prompting enhanced vigilance in hemostasis, meticulous stapling technique, and anticipatory postoperative management.

This study represents the first identification of preoperative anemia as a non-negligible independent risk factor in the context of uniportal VATS pulmonary resection. Anemia signifies impaired blood oxygen-carrying capacity. Adequate tissue oxygen supply is essential for all phases of wound healing, including the inflammatory, proliferative, and remodeling stages. Pre-existing anemia creates a background “oxygen debt,” which surgical trauma further exacerbates by worsening the oxygen supply-demand imbalance. This condition may directly suppress fibroblast activity and collagen synthesis, consequently delaying the healing process at both the pleural surface and pulmonary parenchymal margins. Such delayed healing prevents the rapid restoration of equilibrium between pleural fluid production and absorption. This finding highlights the importance of preoperative identification and active correction of anemia as a crucial modifiable target for optimizing preoperative patient condition and improving postoperative outcomes.

The sensitivity analysis further enriched the clinical value of these risk factors. The combined prediction model demonstrated excellent discriminative performance (AUC = 0.892), significantly surpassing any single indicator, strongly supporting the comprehensive assessment of multiple risk factors in clinical practice rather than reliance on individual parameters. Among these, preoperative FEV1% (AUC = 0.812) and intraoperative blood loss (AUC = 0.801) exhibited outstanding predictive capability, suitable for preoperative evaluation and intraoperative risk warning respectively. The number of stapler cartridges used (AUC = 0.758), with its high sensitivity, serves as a practical tool for early postoperative screening of high-risk patients. Although deep nodule resection and preoperative anemia showed relatively limited individual predictive performance, their strong independence in the multivariate model warrants heightened clinical vigilance when present.

The identification of five binary predictors with relatively comparable effect sizes (*β* coefficients ranging from 1.234 to 1.891) facilitates the conceptual translation of our model into a simple, clinically actionable risk score for PPCTD. A practical implementation could involve two stages: (1) a preoperative score utilizing only preoperatively assessable variables (CT-estimated deep-seated nodule, FEV1% < 70%, and anemia) for initial risk counseling and targeted prehabilitation; and (2) a postoperatively updated score that incorporates intraoperative variables (blood loss ≥250 mL and use of ≥4 stapler cartridges) for final risk stratification and guidance of postoperative management. Given the similarity in effect sizes, two straightforward scoring methods are proposed based on our data: Option A (Simple Additive Score): Assign 1 point for the presence of each of the five predictors, yielding a total score of 0–5. Risk categories (e.g., low: 0–1, intermediate: 2–3, high: 4–5) could be defined empirically based on the observed event rates within our cohort. Option B (Weighted Integer Score): Assign points proportionally to the *β* coefficients (e.g., deep nodule resection: 4 points; FEV1% < 70%: 3 points; ≥4 cartridges: 3 points; blood loss ≥250 mL: 3 points; anemia: 2 points), yielding a total score of 0–15, with cut-offs calibrated to predicted probabilities. The *β* coefficients and event rates provided in this study serve as the foundation for such scores. Their formal derivation, optimal threshold determination, and validation of clinical utility represent a vital next step for prospective, multi-center studies.

This study has several limitations inherent to its design and setting. First, as a retrospective, single-center observational cohort analysis, it is susceptible to selection and information bias, and the associations identified cannot establish causality. Second, the predictive model, despite robust internal validation, lacks external validation in an independent cohort, which is necessary to confirm its generalizability to other institutions and patient populations. Third, our findings may be influenced by institution-specific perioperative protocols, particularly standardized chest tube management practices (e.g., criteria and timing for removal, use of suction), which could affect the primary outcome and limit direct comparability with centers using different protocols. Fourth, although our sample size is considerable, it remains insufficient for detailed subgroup analyses (e.g., by specific resection type or cancer stage) and for including all potential confounders, such as detailed analgesic regimens or dynamic drainage trends. Fifth, we developed a single integrative predictive model. While this model is most applicable for postoperative risk assessment, the construction of separate, phase-specific models (e.g., a purely preoperative model for counseling and an intraoperatively-updated model for management)—a methodologically appealing suggestion—was beyond the scope of this study. Future large-scale, multicenter prospective studies are needed to externally validate our model, control for center-specific practices, develop stage-specific predictive tools, and ultimately implement refined risk-stratified clinical pathways. Fifth, due to the retrospective nature of the study, detailed and standardized data on specific sublobar resection techniques (wedge vs. segmentectomy), exact lymphadenectomy templates, and comprehensive pathological subclassifications were not uniformly available for the entire cohort. Consequently, our analysis adjusted for the broad category of resection extent (lobectomy vs. sublobar) but could not fully account for the potential variability within these categories or the nuances of nodal dissection. It is important to recognize that unmeasured heterogeneity in resection extent and technique may act as a confounder and partly explain the observed associations with predictors such as stapler use and blood loss. Therefore, the identified risk factors should be interpreted as valid within the documentation constraints of this single-center dataset, and their independent contribution should be confirmed in future studies with more granular procedural data. Third, our findings may be influenced by institution-specific perioperative protocols, particularly standardized chest tube management practices (e.g., criteria and timing for removal, use of suction), which could affect the primary outcome and limit direct comparability with centers using different protocols. Furthermore, while we provided an evidence-based justification for the >7-day cutoff and performed a sensitivity analysis with an alternative threshold (>5 days), it should be acknowledged that definitions of prolonged drainage vary across institutions and studies, which may affect the comparability of risk estimates.

In summary, while deep nodule resection, use of ≥4 stapler cartridges, blood loss ≥250 mL, preoperative FEV1% < 70%, and anemia were identified as independent risk factors for PPCTD in our cohort, readers should be mindful that these associations are derived from a retrospective analysis with inherent data limitations, including unmeasured variation in specific resection techniques. These predictors provide a useful framework for risk assessment in settings with similar surgical documentation practices, but their generalizability and independent causal roles require validation in prospective studies with standardized, detailed procedural reporting.

## Data Availability

The raw data supporting the conclusions of this article will be made available by the authors, without undue reservation.
